# An Updated Meta-Analysis of RCTs of Colchicine for Stroke Prevention in Patients with Coronary Artery Disease

**DOI:** 10.3390/jcm10143110

**Published:** 2021-07-14

**Authors:** Aristeidis H. Katsanos, Lina Palaiodimou, Christopher Price, Marios Themistocleous, Robin Lemmens, Ioannis Michopoulos, Marios K. Georgakis, Christian Weimar, Peter Kelly, Georgios Tsivgoulis

**Affiliations:** 1 Division of Neurology, Population Health Research Institute & McMaster University, Hamilton, ON L8S 4L8, Canada; 2 Second Department of Neurology, School of Medicine, Attikon University Hospital, National and Kapodistrian University of Athens, 45701 Athens, Greece; lina_palaiodimou@yahoo.gr (L.P.); tsivgoulisgiorg@yahoo.gr (G.T.); 3 Population Health Sciences Institute, Newcastle University, Newcastle NE1 7RU, UK; c.i.m.price@newcastle.ac.uk; 4 Department of Neurosurgery, Pediatric Hospital of Athens, 45701 Athens, Greece; mthemistocleous@gmail.com; 5 Department of Neurosciences, Experimental Neurology and Leuven Research Institute for Neuroscience and Disease (LIND), KU Leuven—University of Leuven, B-3000 Leuven, Belgium; robin.lemmens@uzleuven.be; 6 Center for Brain and Disease Research, Laboratory of Neurobiology, VIB, B-3000 Leuven, Belgium; 7 Department of Neurology, University Hospitals Leuven, B-3000 Leuven, Belgium; 8 Second Department of Psychiatry, Medical School, National and Kapodistrian University of Athens, “Attikon” General Hospital, 45701 Athens, Greece; imihopou@med.uoa.gr; 9 Institute for Stroke and Dementia Research, University Hospital, Ludwig-Maximilians-University, 81377 Munich, Germany; mgeorgakis91@gmail.com; 10 Institute for Medical Informatics, Biometry and Epidemiology, University of Duisburg-Essen, 47057 Duisburg, Germany; christian.weimar@uk-essen.de; 11 BDH-Clinic Elzach, 79215 Elzach, Germany; 12 Health Research Board Stroke Clinical Trials Network Ireland and Mater University Hospital, University College Dublin, D04 V1W8 Dublin, Ireland; pjkelly@mater.ie; 13 Department of Neurology, University of Tennessee Health Science Center, Memphis, TN 38103, USA

**Keywords:** stroke, colchicine, coronary artery disease, prevention

## Abstract

Emerging evidence from randomized controlled clinical trials (RCTs) suggests that colchicine has cardiovascular benefits for patients with coronary disease, including benefits for stroke prevention. We performed an updated systematic review and meta-analysis of all RCTs reporting on stroke outcomes during the follow-up of patients with a history of cardiovascular disease randomized to colchicine treatment or control (placebo or usual care). We identified 6 RCTs including a total of 11,870 patients (mean age 63 years, 83% males) with a mean follow-up of 2 years. Colchicine treatment was associated with a lower risk of stroke during follow-up, compared to that of placebo or usual care (risk ratio = 0.49, 95% confidence interval: 0.31–0.80; *p* = 0.004), without heterogeneity across the included studies (I^2^ = 0%, *p* for Cochran’s Q = 0.52). In the subgroup analysis, no heterogeneity (*p* = 0.77) was identified in the effect of colchicine on stroke prevention between patients with recent acute (RR = 0.55, 95% CI: 0.15–2.05) or chronic stable (RR = 0.43, 95% CI: 0.21–0.89) coronary artery syndromes. In conclusion, we found that colchicine treatment decreases the stroke risk in patients with a history of atherosclerotic cardiovascular disease.

## 1. Introduction

Emerging evidence from randomized controlled clinical trials (RCTs) suggests that colchicine has cardiovascular benefits in patients with a history of coronary artery disease [1,2,3,4]. In our previous systematic review and meta-analysis, we reported a lower risk of stroke outcomes for patients with a history of cardiovascular disease randomized to colchicine treatment [5].

In light of the two recently published RCTs examining the use of low-dose colchicine in patients with atherosclerotic cardiovascular disease (ASCVD) [6,7], we performed an updated systematic review and meta-analysis to improve our current understanding of the effect of colchicine treatment on stroke risk in patients with ASCVD.

## 2. Materials

The present systematic review and meta-analysis is reported according to the preferred reporting items of systematic reviews and meta-analyses (PRISMA) statement.

We searched Medline, Scopus and the Cochrane Central Register of Controlled Trials (CENTRAL) on 15 September 2020 for published RCTs reporting on incident strokes during the follow-up of patients with a history of ASCVD, in colchicine treatment versus placebo or usual treatment care groups. Reference lists of all articles that met the inclusion criteria and of relevant review articles were examined to identify studies that may have been missed by our initial database search. We excluded non-randomized studies, reports not providing incident stroke rates during follow-up and studies performed in patients undergoing surgical procedures [5]. Risk of bias for each included study was assessed with the relevant tool from the Cochrane Collaboration [6]. Literature search and study quality assessment was performed by two independent authors (AHK & LP) and all emerging conflicts were resolved after discussion with a third author (GT).

For each included study, we calculated the corresponding risk ratios (RRs) and 95% confidence intervals (95% CI) for incident stroke during follow-up between patients randomized to colchicine treatment or placebo/usual care. Study estimates were pooled using a random-effects model. Heterogeneity was assessed with the I^2^ and Cochran’s Q statistics. Number needed to treat (NNT) was calculated using the formula NNT = 1/((1-RR) × incident stroke rate in the control groups), as previously described [5]. Due to the limited number of included studies, the risk of publication bias was assessed with a graphical funnel plot inspection. Finally, we performed a subgroup analysis by dichotomizing studies according to the inclusion of patients with acute or stable ASCVD.

All statistical analyses were conducted using the Cochrane Collaboration’s Review Manager (RevMan 5.3) Software Package (Copenhagen: The Nordic Cochrane Centre, The Cochrane Collaboration, 2014).

## 3. Results

**Figure 1:**
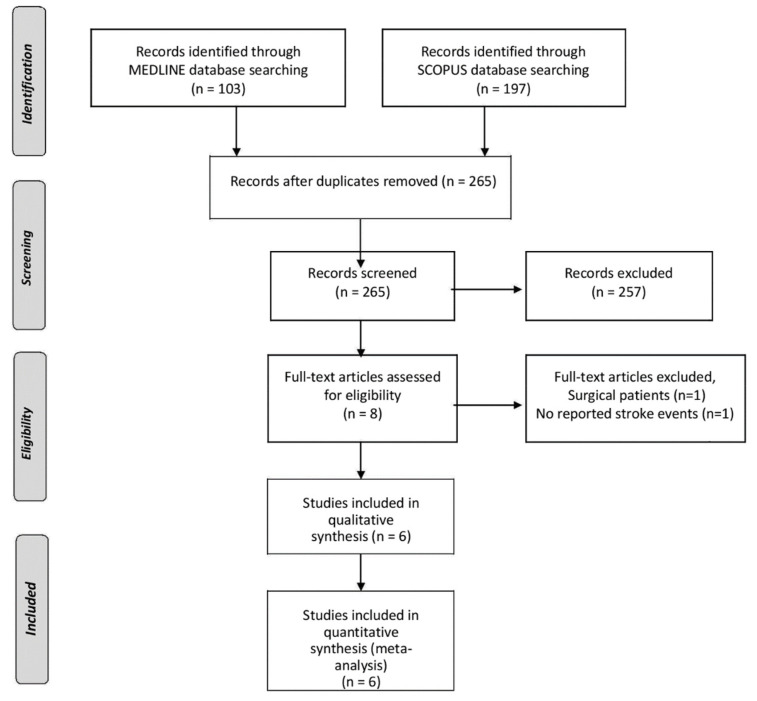
Flow chart presenting the selection of eligible studies.

**Figure 2:**
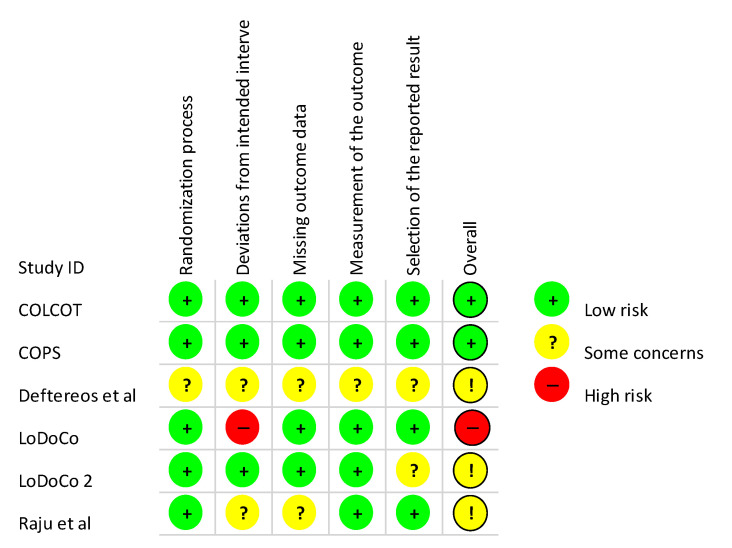
Risk of bias summary that reviews authors’ judgments about each risk of bias item for each included study.

**Figure 3:**
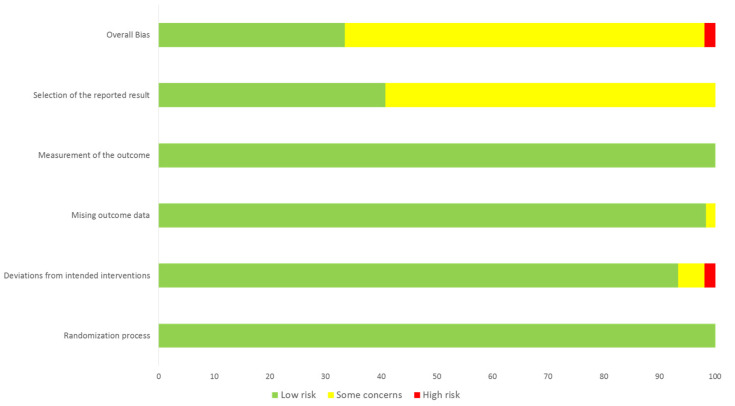
Risk of bias graph that reviews authors’ judgments about each risk of bias item presented as percentages across all included studies.

The literature search in Medline and Scopus databases retrieved 103 and 197 results, respectively (Figure 1). After excluding two study protocols that did not meet our inclusion criteria, we identified 6 RCTs including a total of 11,870 patients (mean age 63 years, 83% males) with a history of ASCVD. The percentage of patients with a history of stroke in the included studies was either low or unknown (Table 1). The risk of selection and performance bias were marked as unclear in one RCT, which did not report sufficiently on the methods of randomization and allocation concealment (Figure 2 and Figure 3) [2]. Detection and attrition bias were considered unclear in two RCTs, reporting no blinding of participants and personnel, and more than a 5% loss to follow-up [2,4]. Reporting bias was considered unclear in two RCTs, due to either a lack of a publicly available protocol [2], or multiple revisions of the study endpoints during the trial [7]. Risk of performance bias was considered high in one RCT that used single blinding of the outcome assessors only [3], whereas all other included studies used additional blinding of participants and study personnel [1,2,4,7,8].

**Figure 4:**
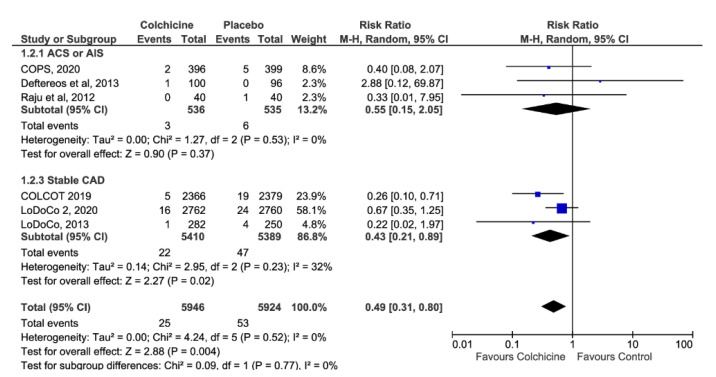
Forest plot on the association of colchicine treatment with the risk of stroke during follow-up in patients with a history of acute or stable cardiovascular disease.

**Figure 5:**
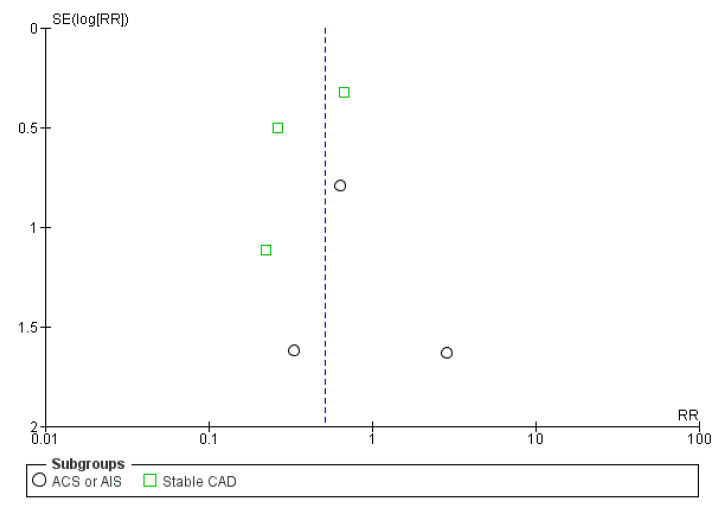
Funnel plot of included studies.

In the overall analysis, colchicine treatment was associated with a lower risk of stroke during follow-up, compared to placebo or usual care (RR = 0.49, 95%CI: 0.31–0.80; *p* = 0.004; Figure 4), without heterogeneity across the included studies (I^2^ = 0%, *p* for Cochran’s Q = 0.52). In the subgroup analysis no heterogeneity (*p* = 0.77) was identified in the effect of colchicine on stroke prevention between patients with acute (RR = 0.55, 95%CI: 0.15–2.05) or stable (RR = 0.43, 95%CI: 0.21–0.89) coronary syndromes. No funnel plot asymmetry was uncovered (Figure 5). Based on the overall risk reduction of 51% and the pooled incident stroke rate across control groups (0.9%) in the included RCTs, daily administration of low-dose colchicine to 218 patients with history of ASCVD would prevent one stroke during an average follow-up interval of 2 years.

## 4. Discussion

Our updated systematic review and meta-analysis supports the accumulating evidence on the benefit of low-dose colchicine for stroke risk reduction in patients with ASCVD. Inflammation has a crucial role in the pathophysiology of atherosclerotic plaque destabilization and thromboembolism, with inflammatory cells being involved in all stages of atherosclerosis development [9,10]. Experimental studies have shown that microscopic cholesterol crystals form in the early stages of atherosclerotic plaque development, and may be a potent inflammatory stimulus for neutrophils and macrophages [11]. Cholesterol crystals activate monocytes and macrophages via the intracellular Nod-like receptor protein 3 (NLRP 3) inflammasome protein complex, resulting in increased interleukin-1β (IL-1β) expression [12], which acts as a key mediator of the initiation of local and systemic inflammatory cascades [13,14]. Expression of cytokines and collagenolytic enzymes, such as metalloproteinases from macrophages and other cells, contributes to erosion and rupture of the fibrous cap, which results in the exposure of circulating platelets and coagulation factors to the pro-thrombotic core, and subsequent thromboembolism [10]. Colchicine could thus constitute a new and important treatment for secondary prevention after stroke, by targeting inflammation via pleiotropic actions, including the inhibition of interleukin-1β (IL1-β) and IL-6 synthesis, and the reduction of microtubule-dependent leucocyte motility and mitosis [15].

Compared to our previous meta-analysis [5], the present report incorporates data from two recently published RCTs [7,8], increasing the sample size of the pooled analyses by more than two-fold (from 5553 to 11870 total patients). In our updated meta-analysis, we performed a quality control of the included studies using the newly developed risk of bias tool from the Cochrane Collaboration [6]. Moreover, by incorporating these two newly published RCTs [7,8] and therefore additional statistical power, this allowed us to perform a subgroup analysis to evaluate the potential disparity in the treatment effect of colchicine, according to the stage of ASCVD (acute versus stable). This subgroup analysis provided no evidence for a differential treatment effect of colchicine on stroke prevention between patients enrolled, due to acute or stable ASCVD. This observation supports a long-term anti-inflammatory effect for colchicine, and may be used to inform the design of future trials.

Despite the strengths of our work, we emphasize that these results need to be interpreted with caution, as patients with prior stroke were under-represented, and the inclusion criteria, dosage, and follow-up duration varied between included studies. Despite these differences, no evidence of statistical heterogeneity was detected in the analyses. The unknown amount or under-representation of patients with a history of previous stroke is another point that requires additional consideration. Moreover, it should be noted that no safety endpoints were addressed in the present systematic review and meta-analysis. Of note, there was a higher incidence of death from non-cardiovascular causes in the colchicine group, compared to placebo in the LoDoCo2 trial [7], and this was also speculated in the COPS trial [8]. Finally, information on stroke type and mechanisms are not reported within included studies, and therefore the potential effect of colchicine on diverse stroke mechanisms remains unknown.

Colchicine is a particularly attractive treatment candidate, particularly for patients with a recent stroke, as it is unlikely to increase the risk of intracranial or extracranial bleeding in this vulnerable patient population. The utility of low-dose colchicine (0.5 mg/day) for the prevention of major vascular events following mild ischemic stroke or high-risk TIA is currently being evaluated in the Colchicine for Prevention of Vascular Inflammation in Non-Cardio Embolic stroke (CONVINCE) trial [16]. If colchicine is proven to be safe and effective, then this low-cost approach can have the potential to change clinical practice and improve the health outcomes of ischemic stroke survivors.

## Figures and Tables

**Table 1:** Characteristics of included studies.

Study Name	Population	Number of Patients	Dose	Median Follow-Up	Age (Years)	Males	Smoking	HTN	DM	History of ASCVD	History of Stroke/TIA
COLCOT, 2019 [1]	MI within 1 month	4745	0.5 mg OD	22.6 months	60.6 ± 10.7	81%	30%	51%	20%	16%	3%
COPS, 2020 [8]	ASCVD	795	0.5 mg BID (1 month)/0.5 mg OD (11 months)	12 months	59.8 ± 10.3	79%	35%	50%	19%	15%	2%
Deftereos et al., 2013 [2]	Diabetics undergoing PCI	196	0.5 mg BID	6 months	63.6 ± 7.0	65%	38%	49%	100%	31%	N/A
LoDoCo, 2013 [3]	ASCVD	532	0.5 mg OD	36 months	66 ± 9.2	89%	5%	N/A	30%	23%	N/A
LoDoCo 2, 2020 [7]	ASCVD	5522	0.5 mg OD	28.6 months	66 ± 8.6	85%	12%	51%	18%	84%	N/A
Raju et al., 2012 [4]	ASCVD or AIS	80	1 mg OD	1 month	57.2 ± 10.0	89%	79%	43%	16%	18%	4%

ASCVD: atherosclerotic cardiovascular disease, AIS: acute ischemic stroke, PCI: percutaneous coronary intervention, OD: once daily, BID: twice daily, HTN: hypertension, DM: diabetes mellitus, TIA: transient ischemic attack.

## Data Availability

All data used for analyses is available within the manuscript and the original publications of the included studies.
